# N-methyl-N-nitrosourea-induced cerebellar hypoplasia in rats: Effect of arachidonic acid supplementation during the gestational, lactational and post-weaning periods

**DOI:** 10.3892/etm.2013.1219

**Published:** 2013-07-12

**Authors:** KATSUHIKO YOSHIZAWA, YUKO EMOTO, YUICHI KINOSHITA, TAKASHI YURI, AIRO TSUBURA

**Affiliations:** 1 Department of Pathology II, Kansai Medical University, Hirakata, Osaka 573-1010, Japan; 2 Division of Pathology, Kansai Medical University Takii Hospital, Moriguchi, Osaka 570-8507, Japan

**Keywords:** arachidonic acid, brain, cerebellar hypoplasia, N-methyl-N-nitrosourea

## Abstract

Arachidonic acid (AA) is a fatty acid that is important for visual and brain development and is commonly added as a functional food ingredient to commercial infant formulas worldwide. However, few studies have examined whether AA supplementation during neonatal life has an effect on neuronal abnormalities. In the present study, the effect of dietary AA supplementation in dams during gestation and lactation was investigated by examining N-methyl-N-nitrosourea (MNU)-induced cerebellar hypoplasia in young Lewis rats. Dams were fed a 2.0% AA diet or a basal diet (<0.01% AA). At birth (postnatal day 0), male and female pups received a single intraperitoneal injection of 35 mg/kg MNU or vehicle. Brain weights were measured and a morphological analysis of macroscopic and histological specimens was conducted after 7, 14, 21, 28 and 60 days. Irrespective of whether the rats had been fed an AA diet, the brain weights of the MNU-treated rats, particularly the weights of the cerebellum, were decreased compared with those of the MNU-untreated rats from the 14th day following the MNU injection. Macroscopic reductions in the cerebellar length and/or width and histologically observed reductions in cerebellar vertex height and/or cortex width were also detected in the MNU-treated rats, irrespective of whether the rats had been fed with AA. Histopathologically, the MNU-treated rats (irrespective of AA supplementation) exhibited disorganization of the cerebellar cortex and disarrangement of the cortical layers (loss and/or disturbance of the molecular, Purkinje and granular cell layers). There were no significant differences in any parameters among the MNU-treated rats, irrespective of whether the rats had been fed an AA diet. In conclusion, an AA-rich diet for dams during gestation and lactation did not modify MNU-induced cerebellar hypoplasia in their offspring.

## Introduction

The brain is a highly organized organ that is responsible for learning, memory, emotion and social behavior. The frequency of cerebellar damage as a complication of premature birth is increasing (1). Cerebellar hypoplasia is a developmental disorder characterized by the incomplete development or underdevelopment of the cerebellum; this disorder may be focal or diffuse/generalized (2). In infancy, the symptoms of cerebellar hypoplasia include developmental delay, hypotonia, ataxia, seizures and involuntary eye movements (nystagmus). At later ages, symptoms include headache, vertigo, imbalance and hearing impairment. There is no standard course of treatment for cerebellar hypoplasia, and only symptomatic and supportive therapies are provided. Gestational exposure to drugs (such as nicotine, cocaine, ethanol, glucocorticoids, phenytoin and anticancer drugs) and radiation (including X-rays) during gestation may induce cerebellar abnormalities in animals and/or humans (1,3–5).

N-methyl-N-nitrosourea (MNU), an alkylating agent, is a potent chemical genotoxic carcinogen (6). MNU induces cancers of the breast, gastrointestinal tract, respiratory tract, lymphoreticular tissue, skin, teeth, pancreas and kidney, depending on the route and timing of exposure and the animal strain (7–10). MNU has been widely used to induce neural toxicity and tumors in animal models (11), due to the fact that it crosses the blood-brain barrier (12,13). MNU causes O^6^-methylguanine-induced point mutations, which have been suggested to be responsible for the initiation of carcinogenesis (14) and neuronal damage during gestational exposure (15,16). MNU exposure during the prenatal/neonatal period induces two types of brain hypoplasia: Microcephaly (hypoplasia of the cerebral cortex) is the result of fetal mouse exposure to MNU on day 13 or 15 of the gestation period (6,17), while cerebellar hypoplasia is the result of neonatal rat exposure to MNU (18–20).

Arachidonic acid (AA) is a polyunsaturated fatty acid present in the phospholipids of cell membranes, and it is particularly abundant in the retina and brain (21,22). Neurological health requires sufficient levels of docosahexaenoic acid (DHA) and AA (23). Early infancy may be a critical period when visual and brain developments are susceptible to the effects of inadequate stores or a deficient intake of DHA and AA (24). AA drives postnatal neurogenesis and elicits a beneficial effect on prepulse inhibition in Pax6 knockout rats, characterized by impaired postnatal neurogenesis (25,26). Randomized clinical trials of supplemental DHA and AA have been conducted in full-term infants, and infants who received the supplementation demonstrated enhanced cognitive functions, as compared with the control groups (27,28). MNU has been demonstrated to induce retinal damage due to the selective formation of the DNA adduct, 7-methyldeoxyguanosine, in photoreceptor cell nuclei followed by photoreceptor cell apoptosis (29,30), while AA supplementation during the gestational, lactational and post-weaning periods has been shown to prevent MNU-induced retinal degeneration in young rats (31).

The aim of the present study was to elucidate the effect of prenatal and postnatal dietary AA supplementation on MNU-induced cerebellar hypoplasia in young Lewis rats.

## Materials and methods

### Animal procedures

The study protocol and all animal procedures were approved by the Animal Care and Use Committee of Kansai Medical University (Hirakata, Japan) and were in accordance with the guidelines for animal experimentation at Kansai Medical University. Sixteen 10-week-old female SPF/VAF rats (LEW/CrlCrlj) that were 1-week pregnant were purchased from Charles River Japan (Yokohama, Japan). The rats were maintained in specific pathogen-free conditions and had free access to water and CE-2-modified diets containing different doses of AA. Animals were housed in plastic cages with paper-chip bedding (Paper Clean; Japan SLC Inc., Hamamatsu, Japan) in an air-conditioned room at 22±2ºC and 60±10% relative humidity with a 12-h light/dark cycle. Offspring were sacrificed to leave a maximum of 10 per dam, and the dams were maintained on their respective diets during the 21-day lactation period. During a post-weaning period of up to 60 days, the offspring were maintained on a CE-2 diet. A total of 115 male and female pups were used in this study. Four to ten rats were sacrificed at each time point (7, 14, 21, 28, and 60 days), and similar numbers of males and females in each dietary group were included.

### Chemical and dose formulation

MNU was obtained from Sigma-Aldrich (St. Louis, MO, USA) and was kept at −80ºC in the dark. The MNU solution was dissolved in physiological saline containing 0.05% acetic acid immediately prior to use. MNU (35 mg/kg) or vehicle (physiological saline containing 0.05% acetic acid) was administered by intraperitoneal (i.p.) injection.

### AA-supplemented diet

As in previous studies, the AA-supplemented diet was formulated by CLEA Japan, Inc. (Tokyo, Japan) (9,10,31). AA was purchased from Cargill Alking Bioengineering LLC (Wuhan and Hubei, China). The diet with 2.0 w/w% AA was semi-purified based on the modified CE-2 formulation (CLEA Japan), while the basal diet consisted of modified CE-2. Gas chromatography analyses of the fatty acid compositions of the diets have been previously reported (10). The total fatty acid volumes were 47.20, 86.75 and 126.63 μg/mg diet for the CE-2 diet (0.006 w/w% AA), basal diet (0.008 w/w% AA), and 2.0% AA diet, respectively. The diets were stored at 4ºC to prevent lipid oxidation prior to use.

### Experimental procedures

**Figure 1:**
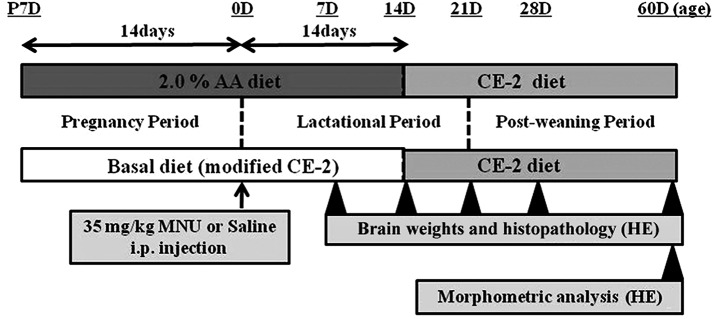
Schema of experimental protocol. MNU, N-methyl-N-nitrosourea; AA, arachidonic acid; i.p., intraperitoneal; HE, hematoxylin and eosin.

**Figure 2:**
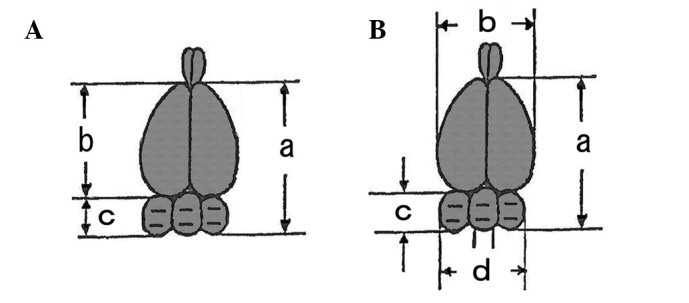
Evaluation of brain weight and gross morphometry. (A) The (a) total brain weight; (b) cerebral weight and (c) cerebellar weight including the medulla oblongata were determined. The method was modified from a previous study [Ogura *et al*, (4)]. (B) Gross morphometric measurements of (a) total brain length (from the rostral border immediately lateral to the most caudal border of the cerebellum); (b) cerebral width; (c) cerebellar length (over the middle of the vermis) and (d) cerebellar width were taken. The method was modified from previous studies (Ogura *et al*, (4); Bolon *et al*, (32)].

Male and female Lewis rats were fed with the basal diet or an experimental diet (2.0% AA) from fertilization to sacrifice. At birth (0 days of age), the rats received an i.p. injection of vehicle (physiological saline) or 35 mg/kg MNU (Fig. 1). At 7, 14, 21, 28, and 60 days following MNU or vehicle treatment, rats were anesthetized with isoflurane (Forane^®^; Abbot Japan Co., Ltd., Tokyo, Japan) and sacrificed by exsanguination from aortic transection. The time-points were predominantly based on guidelines for neuropathological assessment in developmental neurotoxicity testing (32). During the experiment, all pups were observed daily for clinical signs of toxicity and were weighed at the time of MNU treatment and on the day of sacrifice. Brains were quickly removed at the time of sacrifice, and complete necropsies were conducted on all animals to check for systemic toxicities induced by AA supplementation. Brain weights (cerebrum and cerebellum with medulla oblongata) were measured separately (Fig. 2A) by a method similar to a previous study (4). The food consumption and body weight of the dams were measured once per week to estimate the actual dosage of AA during the pregnancy and lactation periods.

### Macro- and histopathological examinations

**Figure 3:**
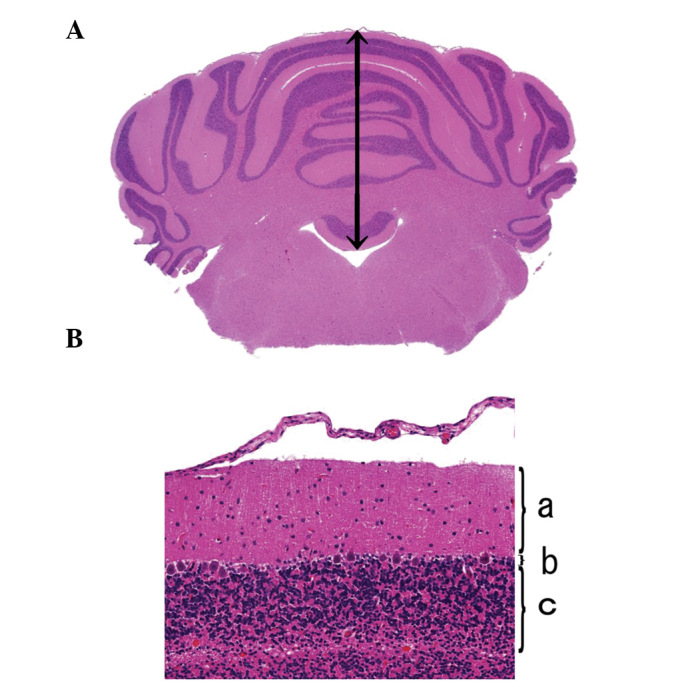
Qualitative linear measurements of the cerebellum. (A) Total height, and (B) the widths of the (a) molecular, (b) Purkinje and (c) granular cell layers at the cerebellar vertex. This method was modified from those in previous studies [Biran *et al*, (1); Bolon *et al,*(32)]. The cerebellum was that of a normal saline-treated rat fed a basal diet, at the age of 60 days. Hematoxylin and eosin staining.

Brain tissues were fixed overnight in 10% neutral buffered formalin, embedded in paraffin, sectioned at a thickness of 4 μm and stained with hematoxylin and eosin (HE). Following fixation, macroscopic photographs were taken of all brains, and the total brain length (from the rostral border immediately lateral to the most caudal border of the cerebellum), cerebral width, cerebellar length (over the middle of the vermis) and cerebellar width were measured with a ruler (Fig. 2B) by a method modified from previous studies (4,32). The gross trimming levels of the brain were levels three and five for the cerebrum and level seven for the cerebellum with medulla oblongata, in accordance with the recommendation for neuropathological assessment in developmental neurotoxicity testing (32). HE-stained sections of the brains were scanned with a high-resolution digital slide scanner (NanoZoomer 2.0 Digital Pathology; Hamamatsu Photonics, Hamamatsu, Japan) to prepare the digital images. The image files were opened in color mode with NDP.view software (Hamamatsu Photonics). Qualitative linear measurements of the cerebellum were obtained in order to determine the height of the cerebellum (Fig. 3A) and the widths of the molecular, Purkinje and granular cell layers at the cerebellar vertex (Fig. 3B), using methods modified from previous studies (1,32).

Histopathological and morphometrical evaluations were performed by a toxicologic pathologist (K.Y.) certified by the Japanese Society of Toxicologic Pathology and the International Academy of Toxicologic Pathology. The histopathological terminology and diagnostic criteria of rodent nervous lesions were based on the guidelines of the International Harmonization Nomenclature and Diagnostic Criteria for Lesions in Rats and Mice Project (33).

### Statistical analysis

All discrete values are expressed as the mean ± standard error (SE) and were analyzed using the two-tailed independent Student's t-test for unpaired samples, subsequent to confirming the homogeneity of variances. The results include comparisons between MNU- and saline-treated rats fed each diet and between the basal diet-fed rats and rats fed an AA-supplemented diet in the MNU-treated and vehicle-treated groups. P<0.05 was considered to indicate a statistically significant difference.

## Results

### General remarks

No deaths occurred, and no clinical signs or symptoms were evident in any dams during the experimental period. The AA diet did not affect the body weight gain (the growth rate) in pups or result in weight changes in the dams, irrespective of MNU treatment; however, the growth rate in the MNU-treated pups tended to be lower than that in the vehicle-treated pups from the age of 21 days (Table I). Hypoactivity in the open field and poor neuromuscular ability in pole climbing in the cages were observed only in the MNU-treated rats fed a basal or AA diet (data not shown).

### Estimated intake of AA

During the pregnancy and lactation periods, the AA intake of the dams was 4.7 and 9.4 mg/kg/day in the basal diet group, 77.7 and 242.6 mg/kg/day in the 0.1% AA group, 261.8 and 874.0 mg/kg/day in the 0.5% AA group and 1,075.1 and 3,058.5 mg/kg/day in the 2.0% AA group, respectively.

### Brain weights

In the saline-treated rats fed with or without AA, the total weight, cerebrum weight and cerebellum weight increased as the age of the rats increased, which was suggestive of a normal growth rate. Fourteen days subsequent to the MNU treatment, the total weight, cerebrum weight and/or cerebellum weight were significantly decreased compared with those in the saline-treated rats (Table II). There were no significant differences in any parameters between the MNU-treated rats fed with or without AA at the age of 60 days. The decreased growth rates in the cerebrum and cerebellum at the age of 60 days resulted in those structures comprising 80 and 20% of total brain weight in the saline-treated rats fed a basal diet, 83 and 17% in the MNU-treated rats fed a basal diet, 79 and 21% in the saline-treated rats fed an AA diet and 84 and 16% in the MNU-treated rats fed an AA diet, respectively (Table II). These results suggest that the change in brain weight in the MNU-treated rats was due to the significantly reduced weight of the cerebellum.

### Macroscopic analysis of the brains

**Figure 4:**
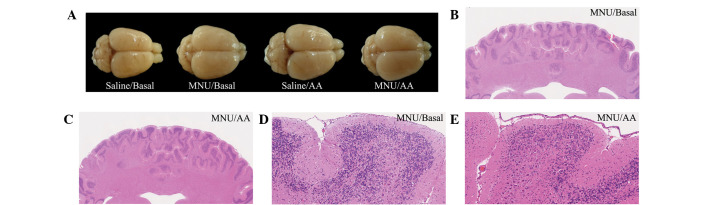
Macroscopic and histopathological brain lesions in rats treated with 35 mg/kg N-methyl-N-nitrosourea (MNU) or saline only at birth. (A) Dorsal views of brains from 28-day-old rats. Left end, saline-treated control rat fed the basal diet (Saline/Basal); second from the left, MNU-treated rat fed the basal diet (MNU/Basal); right middle, saline-treated control rat fed the arachidonic acid (AA)-rich diet (Saline/AA); far right, MNU-treated rat fed the AA-rich diet (MNU/AA). Note the reduction in the cerebellar cortex in the specimens from MNU-treated animals, the disappearance of the cerebellar vermis tubercle and the appearance of quadrigeminal bodies. (B-E) Histopathological lesions of the cerebellum in the MNU/Basal (B and D) and MNU/AA (C and E) rats. Hypoplasia of the cerebellar cortex in MNU-treated rats fed each diet (AA or basal) [B and C; hematoxylin and eosin (HE) staining; magnification ×13]. Disorganization of cerebellar cortex, characterized by the disarrangement of cortical layers and loss and/or disturbance of the molecular, Purkinje and granular cell layers (D and E; HE staining; magnification, ×100).

In the saline-treated rats, irrespective of whether the rats had been fed AA, no brain abnormalities (including in the cerebellum) were observed at any time-point. By contrast, macroscopic abnormalities of the cerebellum were identified in the MNU-treated rats from 21 days subsequent to treatment, irrespective of whether the rats had been fed AA. These abnormalities were characterized by a reduction of the cerebellar vermis tubercle, followed by the altered appearance of quadrigeminal bodies (Fig. 4A).

Morphometrical analysis of the macroscopic brain lesions comprised assessment of the total brain length (from the rostral border immediately lateral to the most caudal border of the cerebellum), cerebral width, cerebellar length (over the middle of the vermis) and cerebellar width at 60 days subsequent to MNU treatment (Table III). In the saline-treated rats fed the AA-rich diet, every parameter examined was consistent with that in the saline-treated rats fed a basal diet. In the MNU-treated rats, the total brain length, cerebellar length and cerebellar width were significantly decreased compared with those in the saline-treated rats (Table III), with measurements of 17,669, 2,534 and 10,804 μm in the MNU-treated rats fed the basal diet and 17,758, 2619 and 11,499 μm in the MNU-treated rats fed the AA diet, respectively. There were no significant differences in any parameters between the MNU-treated rats fed with or without AA. These results suggest that the reduction in the total brain length of the MNU-treated rats was due to the significantly decreased length and width of the cerebellum.

### Histopathological examination of the cerebellum

The histological studies revealed no abnormal changes in the brain (including the cerebellum) at any time-point in the vehicle-treated rats fed with basal or AA diets (data not shown). The external (embryonic) granular cell layer was located on the surface area of the cerebellum in the two groups until the age of 14 days. In the cerebellum of the 21-day-old rats, the external granular cell layer disappeared, followed by the occurrence of the normal development of three cell layers: the molecular, Purkinje and granular cell layers. This suggests the mature development at this age to be a suitable substrate for the majority of the routine methods used in neuropathological evaluation (32). MNU-treated rats fed a basal or AA diet from the age of 7 days exhibited disorganization of the cerebellar cortex, including disarrangement of external granular, Purkinje and inner granular cells (data not shown). A reduced cellularity of the inner granular cell layer and a disperse deposition of Purkinje cells in the inner granular cell layer were observed, followed by thinning of the cerebellar cortex due to loss and/or disturbance of the molecular, Purkinje and granular cell layers, diagnosed as hypoplasia of the cerebellar cortex. At the age of 60 days, the severity of the hypoplasia of the cerebellar cortex in the MNU-treated rats fed a basal diet (Fig. 4B and D) was similar to that in the MNU-treated rats fed an AA-rich diet (Fig. 4C and E).

To confirm the qualitative differences among the treated groups at the age of 60 days, the cerebellar height and the widths of the molecular, Purkinje and granular cell layers at the cerebellar vertex were measured (Table IV). In the saline-treated rats fed an AA-rich diet, every parameter examined was consistent with that in the saline-treated rats fed a basal diet. In the MNU-treated rats, the total height and all parameters of the cortical width (molecular, Purkinje and granular cell layers) were significantly decreased as compared with those in the saline-treated rats (Table IV), with measurements of 1,997.3, 98.2, 9.0 and 137.2 μm in the MNU-treated rats fed a basal diet and 2,062.9, 106.9, 9.3 and 145.6 μm in the MNU-treated rats fed an AA diet, respectively. There were no significant differences in any parameters examined among the MNU-treated rats, irrespective of whether the rats had been fed with AA.

Furthermore no changes in macroscopic or histopathological characteristics were observed in the cerebrum at any time-point in the MNU-treated rats fed a basal diet or AA diet (data not shown).

## Discussion

The present study examined the effects of dietary AA supplementation during the gestational, lactational and post-weaning periods on MNU-induced cerebellar hypoplasia in young rats. Irrespective of whether the rats had been fed an AA diet, the brain weights of the MNU-treated rats, particularly the weights of the cerebellum, were decreased compared with those of the MNU-untreated rats from the 14th day following the MNU injection. Macroscopic reductions in the cerebellar length and/or width and histologically observed reductions in the cerebellar vertex height and/or cortical width were also detected in the MNU-treated rats, irrespective of whether the rats had been fed with AA. Histopathologically, the MNU-treated rats (irrespective of AA supplementation) exhibited disorganization of the cerebellar cortex and disarrangement of the cortical layers (loss and/or disturbance of the molecular, Purkinje and granular cell layers). There were no significant differences in any parameters of the MNU-treated rats fed with or without AA.

MNU exposure during the prenatal period induces two types of brain hypoplasia: microcephaly and cerebellar hypoplasia. Microcephaly (cerebral cortex hypoplasia) has been shown to occur in the offspring of mice intraperitoneally injected with 10 mg/kg MNU on day 13 or 15 of the gestation period (6,17). MNU induces excessive cell death of neural precursor/stem cells and the defective development of the cerebral cortex, resulting in cerebral abnormalities. Embryos during the organogenetic periods of the central nervous system are sensitive to temporal and spatial environmental factors, since these factors affect critical developmental processes, such as proliferation, migration, differentiation, synaptogenesis, myelination and apoptosis (34). Late-onset cerebellar degeneration followed by hypoplasia has been shown to occur in the offspring of mice exposed to 1 mg/kg MNU on day 16 of gestation (19,20). In additional, daily subcutaneous injections of 12.9 mg/kg MNU in rats at the ages of 4–7 days have been demonstrated to induce cerebellar hypoplasia with reduced cellularity of the internal granular cell layer and a disperse deposition of Purkinje cells in the granular cell layer at 14 days subsequent to birth; however, no lesions in the cerebrum were induced (18). Cerebellar hypoplasia is associated with MNU-induced cell death and inhibited cell mitosis in the developing brain, particularly in the cerebellum at the mitotic stage (35). Motor dysfunctions are induced by imbalanced output activities from Purkinje cells to motor neurons. Cerebellar neurons are generated in two germinative neuroepithelia in two waves of proliferation and migration in rats (1). The development stage at day 0 in rats shows the differentiation of Purkinje cells and the second wave genesis and migration of granular cells (1). As indicated in the previously mentioned studies, the target position of brain abnormalities induced by MNU exposure may depend on the exposure period at fetal or neonatal life. Cerebral hypoplasia occurs with MNU exposure at the developmental period of cerebral neurons, while cerebellar hypoplasia occurs with MNU exposure at the period with the most proliferative activity of cerebellar neurons (1,34). Therefore, the present experimental protocol with exposure at birth was a reasonable strategy for MNU to induce cerebellar hypoplasia, but not cerebral anomalies, in rats.

AA, together with DHA, is a fatty acid that is important in central nervous system development; AA is commonly added as a functional food ingredient to commercial infant formula worldwide, in accordance with the international standards of Codex Alimentarius (36). AA and DHA have a critical function in neurodevelopment and the response to neural injury in the neonatal stage (24). The levels of fatty acids in brain tissue may be modified by dietary fatty acid intake (21,37). AA directly affects neural stem/progenitor cells and promotes postnatal neurogenesis (38). Furthermore, AA ameliorates the prepulse inhibition relevant to psychiatric disorder models, such as methylazoxymethanol-treated rats and Pax6 knockout rats, through augmented postnatal neurogenesis (25,26). By contrast, AA exhibits biphasic actions in cultured brain neurons within a narrow concentration range, with induction of cell death on one hand and promotion of cell survival and enhancement of neurite extension on the other (39). The neurotoxic action is mediated by free radicals generated by AA metabolism, whereas the neurotrophic actions are exerted by AA itself (39,40). Dietary AA supplementation may be beneficial as a potential means to delay the onset and/or progression of neural disease by the inhibition of neuronal cell death at narrow windows of susceptibility (in the developmental phase) for neuronal rescue. Although the present strategy of AA supplementation during the gestational, lactational and post-weaning periods has been shown to prevent retinal degeneration in young rats (31), an identical therapeutic approach did not rescue MNU-induced cerebellar hypoplasia in the present study.

In the neurotoxicity model induced by MNU, significant increases in the levels of lipid peroxidation, peroxide products and reactive oxygen species production in the brain (11) have been observed. MNU enhances cellular oxidative stress and induces apoptosis. The antioxidant, butylated hydroxytoluene, is capable of retarding the cerebellar degeneration induced transplacentally by a single injection of 1 mg/kg MNU on day 16 of pregnancy (20), while curcumin, another antioxidant, is capable of rescuing functional and structural changes in the cerebrum of young mice treated with 10 mg/kg MNU (11). An AA-rich diet may have low potency to inhibit or protect the production of cellular oxidative stress in the brain induced by MNU.

The AA intake by Japanese infants via breast milk is ~14.3 mg/kg/day (41). The 2.0% AA diets in the present study provided an AA dose of 1,477 mg/kg/day during pregnancy and 1,876 mg/kg/day during lactation, which represented ~103- and 131-fold, respectively, the quantities consumed by human infants. In combination, the results of the present study indicated that an AA-enriched diet in the prenatal and postnatal periods was unlikely to prevent cerebellar hypoplasia in human infants, despite the importance of AA in brain development. Further studies with other animal models are required in order to understand any effects of AA on cerebellar hypoplasia.

## Figures and Tables

**Table I:** Sequential changes in body weight (mg).

	Days after treatment				
	
Group	7	14	21	28	60
Basal diet^a^ + saline injection	15.3	31.4	48.3	78.3	254.3
Basal diet + MNU injection	13.1	28.1	29.3^c^	44.4^c^	133.4^c^
AA diet^b^ + saline injection	14.5	33.3	48.1	83.1	263.3
AA diet + MNU injection	12.1	28.0	29.5^c^	35.5^c^	130.0^c^

a The basal diet contained 0.008% arachidonic acid (AA).

b The AA diet contained 2.0% AA.

c P<0.01 compared with the saline treatment group fed with the same diet. Mean values were significantly different between saline and N-methyl-N-nitrosourea (MNU) treatment in each diet group. By contrast, mean values were not significantly different between the basal and AA diet groups for each treatment.

**Table II:** Sequential changes in brain absolute weight in rats following 35 mg/kg MNU treatment.

A. Basal diet^a^ + saline injection					

	Days after treatment				
	
Brain region	7	14	21	28	60
Total [mg (%)^c^]	744.0	1402 (100)	1597.8 (100)	1930.2 (100)	2297.3 (100)
Cerebrum [mg (%)]	NE	1073.7 (76)	1272.6 (78)	1537.4 (80)	1826.6 (80)
Cerebellum^d^ [mg (%)]	NE	328.3 (24)	325.2 (22)	392.8 (20)	470.7 (20)

B. Basal diet + MNU injection					

	Days after treatment				
	
Brain region	7	14	21	28	60

Total [mg (%)]	628.0^f^	1203.0 (100)	1243.8 (100)^f^	1706.6 (100)^f^	2054.3 (100)^e^
Cerebrum [mg (%)]	NE	963.3 (80)	993.2 (80)^f^	1419.6 (83)	1711.0 (83)^e^
Cerebellum [mg (%)]	NE	239.7 (20)^f^	250.6 (20)^f^	287 (17)^f^	343.3 (17)^f^

C. AA diet^b^ + saline injection					

	Days after treatment				
	
Brain region	7	14	21	28	60

Total [mg (%)]	730.5	1362.7 (100)	1734.8 (100)^g^	1997.5 (100)	2421 (100)
Cerebrum [mg (%)]	NE	1053.0 (77)	1377.6 (79)^g^	1598.8 (80)	1907 (79)
Cerebellum [mg (%)]	NE	309.7 (23)	357.2 (21)	398.8 (20)	514 (21)

D. AA diet + MNU injection					

	Days after treatment				
	
Brain region	7	14	21	28	60

Total [mg (%)]	593.8^f^	1006.2 (100)^f^	1240.2 (100)^f^	1604.2 (100)	1978.9 (100)^f^
Cerebrum [mg (%)]	NE	806.3 (80)^e^	974.2 (79)^f^	1363.6 (85)^f^	1655.3 (84)^f^
Cerebellum [mg (%)]	NE	199.8 (20)^f^	266.0 (21)^f^	240.6 (15)^e^	323.6 (16)^f^

^a^The basal diet contained 0.008% arachidonic acid (AA); ^b^the AA diet contained 2.0% AA; ^c^percentage of total brain weight; ^d^weight including the medulla oblongata. Mean values were significantly different between saline and N-methyl-N-nitrosourea (MNU) treatment in each diet group (^e^P<0.05 and ^f^P<0.01) and between the basal and AA diet groups for each treatment, (^g^P<0.05). NE, not examined.

**Table III:** Sequential changes in brain length in rats 60 days after 35 mg/kg MNU treatment.

	Brain length (μm)			
	
Group	Total brain length	Cerebral width	Cerebellar length	Cerebellar width
Basal diet^a^ + saline injection	18981	14614	4986	11539
Basal diet + MNU injection	17669^d^	13999	2534^d^	10804^c^
AA diet^b^ + saline injection	19232	14756	5367	12030
AA diet + MNU injection	17758^d^	13882	2619^d^	11499

^a^The basal diet contained 0.008% AA; ^b^the arachidonic acid (AA) diet contained 2.0% AA. Mean values were significantly different between saline and N-methyl-N-nitrosourea (MNU) treatment in each diet group (^c^P<0.05 and ^d^P<0.01). By contrast, mean values were not significantly different between the basal and AA diet groups for each treatment.

**Table IV:** Morphometrical changes in the cerebellar cortex in rats 60 days subsequent to 35 mg/kg MNU treatment.

Groups	Cerebellar vertex (μm)			

	Total height	Cortex width		

		Molecular cell layer	Purkinje cell layer	Granular cell layer
Basal diet^a^ + saline injection	4136.0	145.1	20.8	298.3
Basal diet + MNU injection	1997.3^d^	98.2^d^	9.0^d^	137.2^d^
AA diet^b^ + saline injection	4262.5	153.3	22.9	314.8
AA diet + MNU injection	2062.9^d^	106.9^d^	9.3^c^	145.6^d^

^a^The basal diet contained 0.008% arachidonic acid (AA). ^b^The AA diet contained 2.0% AA. Mean values were significantly different between saline and N-methyl-N-nitrosourea (MNU) treatment in each diet group (^c^P<0.05 and ^d^P<0.01). By contrast, mean values were not significantly different between the basal and AA diet groups for each treatment.

## Abbreviations

AA: arachidonic acid
DHA: docosahexaenoic acid
MNU: N-methyl-N-nitrosourea
